# Searching for health equity: validation of a search filter for ethnic and socioeconomic inequalities in transport

**DOI:** 10.1186/s13643-019-1009-5

**Published:** 2019-04-11

**Authors:** Jamie Hosking, Alexandra Macmillan, Rhys Jones, Shanthi Ameratunga, Alistair Woodward

**Affiliations:** 1 Section of Epidemiology and Biostatistics, Faculty of Medical and Health Sciences, The University of Auckland, Private Bag 92019, Auckland, 1142 New Zealand; 2 Department of Preventive and Social Medicine, University of Otago, Box 56, Dunedin, PO 9054 New Zealand; 3 Te Kupenga Hauora Māori, Faculty of Medical and Health Sciences, The University of Auckland, Private Bag 92019, Auckland, 1142 New Zealand

**Keywords:** Equity, Ethnicity, Socio-economic status, Transport

## Abstract

**Background:**

Efforts to improve health equity should be informed by the best available evidence. However, equity-related research is inconsistently indexed, and uses a variety of terms to describe key concepts, making it difficult to reliably identify all relevant studies. We report the development and validation of a search strategy for studies investigating whether the effects of interventions differ by ethnicity or socio-economic status, using the field of transport and health as an example.

**Methods:**

Adapting previously described methods, we followed four steps: generation of a test set of eligible studies, search strategy development, search strategy validation, and documentation.

**Results:**

Drawing from 12 systematic reviews, supplemented by additional studies identified by experts and colleagues, we identified a test set of 11 studies that met our eligibility criteria. We assigned five studies to a development set, which we used to develop and refine our search strategy. We assigned the remaining six studies to a validation set, against which we tested our final search strategy. The final search strategy identified all studies in both validation and development sets.

**Conclusions:**

The validated search strategy derived in this study facilitates the conduct of systematic reviews and other literature searches investigating whether the effects of interventions differ by ethnicity or socio-economic status and may be further developed in future for other equity-focused searches and reviews.

## Background

It is generally accepted that efforts to improve health should be informed by the best available evidence, and that systematic reviews are an important tool for forming a reliable representation of all available evidence. Evidence-informed practice is just as important for health equity as for other fields, yet there are still relatively few equity-focused systematic reviews [1]. A prerequisite for reviews of this kind, as well as for other research-related activities such as guideline development, is effective literature searching.

Searching for health equity literature can be challenging, and this may explain why systematic reviews with this focus are scant. In particular, search strategies using equity-related text filters to screen out unrelated studies run the risk of missing relevant literature. This is because studies in this field use a variety of terms to describe key concepts (e.g. inequities, inequalities, disparities) and equity terms are not indexed consistently [1]. This issue is particularly salient for systematic reviews, which must use sensitive search strategies to ensure findings are not biased by missing studies.

One approach to reduce the risk of missing studies is to use an unrestricted search strategy that does not include equity filters. However, such a search strategy may have very low specificity, and return simply too many citations to review, making a systematic review not feasible. Another approach is to limit the scope of the search to a narrow subtopic. However, this could lead to a review that identified few or no eligible studies [2] or may exclude other subtopics that are of interest. A third strategy is to carry out a review or overview of systematic reviews (also called umbrella reviews), drawing on equity-relevant papers that were included within previous non-equity specific reviews [3]. However, these overviews may struggle to provide an effective synthesis if different systematic reviews have focused on different populations, outcomes, or quality appraisal methods [4]. They may also fail to include papers published since the relevant systematic reviews were completed. Therefore, these strategies, while useful, may lead to problems with feasibility, scope, or timeliness.

An alternative approach is to use a validated equity-focused search filter that has a low risk of missing relevant studies. Such filters have been developed and validated for some population subgroups [5], but do not cover all important dimensions of health equity. In particular, we are aware of no validated filters for studies addressing socio-economic status or for ethnicity (with the exception of specific indigenous populations in Canada and Australia [6, 7]).

Therefore, in this study, we developed and validated a search filter for studies investigating whether the effects of interventions differ by ethnicity or socio-economic status. We developed and tested this filter in the field of transport, a social determinant of health with important effects on both health and equity, with the goal of producing a validated strategy that could subsequently be applied or adapted in other areas of health. Transport policies and interventions can affect physical activity and air pollution levels, as well as road traffic injury risk, and lead to substantial health impacts [8]. Health effects arising from transport are seldom distributed equally between different social groups, and there is evidence that ethnicity and social deprivation can be associated with a higher burden of transport-related disease [9, 10].

## Methods

We adapted the general approach described by Hausner et al. [11], first generating a test set of eligible studies, followed by development, validation, and documentation of the search strategy.

### Generation of test set of eligible studies

We generated a test set of eligible studies by reviewing the reference lists of systematic reviews of transport interventions. We also contacted experts and colleagues working in this field. Given the absence of comprehensive systematic reviews of transport and equity, we included systematic reviews that were not specific to health equity, and then appraised studies against a set of eligibility criteria (Table 1) in order to identify studies that investigated whether the effects of transport interventions differed by ethnicity or socio-economic status.

**Table 1:** Eligibility criteria for studies of whether the effects of transport interventions differ by ethnicity and socio-economic status

**‘Equity-focused’ criteria**	
Inclusion criteria	
• To be eligible, studies must assess whether the impacts of transport interventions differ by ethnicity and socio-economic status (SES). Studies that report effect estimates stratified by SES or ethnicity are eligible, and studies that report whether or not there is an interaction effect between intervention and the ethnicity/SES variable are eligible.	
• Income, education, employment, and housing tenure are eligible measures at individual level. Area deprivation is an eligible area level measure of SES and may be also used as a proxy for individual SES.	
Exclusion criteria	
• Studies that only assess whether there is confounding by SES/ethnicity (rather than whether intervention effects differ by SES/ethnicity) are not eligible.	
**‘Transport intervention’ criteria**	
Inclusion criteria	
• Studies of transport interventions are eligible. This search focuses on interventions that change the amount (distance or duration) of travel by different modes.	
• Eligible outcome measures include all quantitative measures of health and measures of the amount of travel by different modes (e.g. walking, cycling or car use).	
• Quantitative studies are eligible, including both empirical and modelling studies. Under the ‘empirical’ category, randomised controlled trials, non-randomised controlled trials, controlled before-after (CBA) studies and interrupted time series (ITS) studies are eligible. CBA studies may use geographical exposure to the intervention (e.g. proximity to infrastructure) as a basis for assigning participants to intervention and control groups. ITS studies must include at least two data points before the intervention and one after. In addition, modelling studies that estimate the impact of transport interventions or of specified changes in travel on health outcomes are eligible.	
Exclusion criteria	
• Studies that only describe the implementation of transport interventions, and do not assess changes in the amount of travel, are not eligible.	
• Studies that assess change in outcomes through retrospective self-assessment by participants (e.g. ‘do you cycle more now than before the intervention?’), and do not measure the outcome before and after the intervention, are not eligible.	
• Purely qualitative studies are not eligible.	

### Search strategy development

We developed a MEDLINE search strategy in two parts: a ‘transport intervention’ search strategy and an ‘equity-focused’ search for studies addressing ethnicity or socioeconomic status. Our transport intervention search strategy was adapted from a previous Cochrane systematic review of transport and health [12]. Our equity-focused search strategy was adapted from a previous systematic review investigating socio-economic differences in the effectiveness of nutrition interventions [13]. This draft search strategy was further refined using a trial-and-error approach in which search term combinations were tested in MEDLINE to see if they identified the journal articles in the ‘development set’. We aimed to achieve 100% sensitivity while maximising specificity as far as possible. The final search strategy combined the transport intervention and equity-focused search strategies using the ‘AND’ operator.

### Search strategy validation

We assessed the validity of the final search strategy by testing it against a validation set, following the approach used in Hausner et al. [11]. We recorded which studies in the validation set were identified by the final search strategy and which were not.

## Results

### Generation of test set of eligible studies

We identified 10 systematic reviews of transport interventions through database searches and prior knowledge [12, 14–22]. Experts and colleagues working in the field identified two further systematic reviews [23, 24]. From the studies included in those systematic reviews, we identified 125 potentially eligible journal articles. Experts and colleagues suggested a further 33 journal articles, giving a total of 158 journal articles. We retrieved the full text for all 158 articles and assessed them against the systematic review eligibility criteria, resulting in 11 eligible studies.

This test set of 11 articles was randomly divided into a development set and a ‘validation set’ using random number generation in Microsoft Excel. Five studies were assigned to the development set [25–29] and six studies were assigned to the validation set [30–35].

### Search strategy development and validation

Application of the draft search strategy to the development set identified several additional search terms that were added to the equity-focused search strategy to improve sensitivity. Other search terms were removed in order to increase specificity.

The transport intervention search strategy identified 113,133 citations, and the final equity-focused search strategy identified 618,121 citations. Combining the two search strategies using the ‘AND’ operator yielded 8170 citations (Table 2). This final combined search strategy identified all five studies (100%) in the development set. It was then tested against the validation set, in which it identified all six studies (100%).

**Table 2:** Final search strategy with numbers of citations identified by MEDLINE for each combination of terms

1	(equit* or inequit* or inequalit* or disparit* or equality).tw.	85,758
2	(ethnic* or race or racial* or racis*).tw.	184,410
3	((social* or socio-economic or socioeconomic or economic or structural or material) adj3 (advantage* or disadvantage* or exclude* or exclusion or include* or inclusion or status or position or gradient* or hierarch* or class* or determinant*)).tw.	91,032
4	(health adj3 (gap* or gradient* or hierarch*)).tw.	2377
5	Vulnerable populations/ or socioeconomic factors/ or poverty/ or social class/ or Healthcare Disparities/ or Health Status Disparities/ or Poverty areas/ or Urban population/	241,519
6	(SES or SEP or sociodemographic* or socio-demographic* or income or wealth* or poverty or educational level or level of education or educational attainment or well educated or better educated or unemploy* or home owner* or tenure or affluen* or well off or better off or worse off).tw.	204,500
7	or/1–6	618,121
8	(automobile* or auto use* or car or cars or motori#ed. transport* or motorist* or road user* or motor vehicle* or vkt* or vmt* or vehicle kilomet* or vehicle mile*).tw.	38,786
9	(pedestrian* or cyclist* or bicyclist* or bicycling or bik* or bus or buses or busing or busing or trains or rail or railway orpublic transport* or nonmotori#ed. transport or non-motori#ed. transport or active travel* or active transportation).tw.	29,491
10	motor vehicles/ or walking/ or bicycling/	36,909
11	(travel mode* or mod* shift or travel behav*).tw.	527
12	(transport or transportation or travel* or traffic or road* or congestion or fuel or gasoline or parking or commut*).tw.	469,683
13	or/8–12	549,581
14	(polic* or plan or plans or planning or intervention or experiment or trial or program* or tax* or charg* or initiative* or pric* or subsid* or promot* or campaign* or evaluation or evaluating or implementation or implementing or infrastructure or modelling or modelling or impact assessment or cost-benefit analysis).tw. or health impact assessment/	4,017,557
15	13 and 14	113,133
16	7 and 15	8170

Note: the symbol * is a wildcard

## Discussion

In this paper, we report a validation exercise for a search strategy for studies investigating whether the effects of interventions differ by ethnicity or socio-economic status, using the field of transport and health as an example. Our search strategy, which included equity-focused text word limits for ethnicity and socio-economic status, successfully identified all studies in our development set (*n* = 5) and validation set (*n* = 6) of eligible studies, a sensitivity of 100%. The equity-focused filter also greatly improved specificity, reducing the number of citations returned from > 100,000 (for the unfiltered transport intervention search strategy) to < 10,000.

A limitation of this validation exercise is that there were only 11 eligible studies. It would be desirable to test the search strategy with a larger set of eligible studies and in other fields beyond transport. However, in the absence of other validated search strategy filters for socio-economic status and ethnicity (except for single ethnic groupings [6, 7]), this strategy facilitates the conduct of systematic reviews relating to ethnicity and socio-economic status, particularly in the transport field.

We are aware of no published systematic reviews related to transport interventions and health equity, though one ‘umbrella review’ of other systematic reviews found no primary studies of 20 mph zones and health inequalities [36]. Our validation exercise suggests that there may be relatively few primary studies in this area; using previously published systematic reviews and expert advice, we identified only 11 eligible studies addressing ethnicity or socio-economic status. Our search strategy validation could provide a starting point for systematic reviews that could identify additional studies.

Our search strategy successfully identified all studies in our ‘test set’ of eligible studies, which included studies assessing whether intervention effects differed for different ethnic or socio-economic groups. We did not include studies that only addressed ethnicity/SES as a confounder of intervention effects, so our search strategy should not be used to search for such studies. Studies that adjust for ethnicity/SES as a confounder may be less likely to mention ethnicity or SES in the study abstract, making a sensitive search strategy difficult to design.

## Conclusions

We conclude that it is likely to be very difficult to undertake a systematic review in this field without health equity text word limits, due to the very large number of ineligible citations identified (> 100,000 in our transport intervention search strategy). On the other hand, strategies that use unvalidated health equity text word limits run the risk of failing to identify all eligible studies, since equity-related terms are not indexed consistently. We developed and tested a sensitive search strategy that successfully identified all eligible studies in our test set (100% sensitivity), while greatly improving specificity. This validation exercise may provide a useful basis for future systematic reviews relating to ethnicity or socio-economic status, particularly in the transport field, and our search strategy may be further developed in future for other equity-focused searches and reviews.

## Abbreviations

CBA: Controlled before-after study
ITS: Interrupted time series
SES: Socio-economic status
